# Identifying features of ‘pathological demand avoidance’ using the Diagnostic Interview for Social and Communication Disorders (DISCO)

**DOI:** 10.1007/s00787-015-0740-2

**Published:** 2015-07-30

**Authors:** Elizabeth O’Nions, Judith Gould, Phil Christie, Christopher Gillberg, Essi Viding, Francesca Happé

**Affiliations:** Division of Psychology and Language Sciences, Developmental Risk & Resilience Unit, Clinical, Educational, and Health Psychology Research Department, University College London, London, UK; MRC Social, Genetic and Developmental Psychiatry Centre, Institute of Psychiatry, Psychology and Neuroscience, King’s College London, London, UK; The NAS Lorna Wing Centre for Autism, Bromley, Kent UK; The Elizabeth Newson Centre, Sutherland House Children’s Services (NORSACA), Nottinghamshire, UK; Gillberg Neuropsychiatry Centre, University of Gothenburg, Gothenburg, Sweden

**Keywords:** Autism spectrum disorder (ASD), Pathological demand avoidance (PDA), Pervasive developmental disorder, Diagnostic Interview for Social and Communication Disorders (DISCO)

## Abstract

The term ‘pathological demand avoidance’ (PDA) was coined by Elizabeth Newson to describe children within the autism spectrum who exhibit obsessive resistance to everyday demands and requests (Newson et al., Arch Dis Child 88:595–600, 2003). Clinical accounts describe avoidance strategies including apparently strategic use of distraction or socially shocking behaviour, and obsessive need for control, reflected in domineering behaviour to peers and adults. Educational and management approaches effective for PDA reportedly differ from those for ‘typical’ autism spectrum disorders (ASD), and include novelty, humour and flexibility. Identification of PDA in individuals with ASD may have important implications for management (Eaton and Banting, J Learn Disabil Offending Behav 3:150–157, 2012). Despite increasing interest, no clinician-rated instrument for PDA has been developed. Here, items relevant to PDA were identified from the Diagnostic Interview for Social and Communication Disorder (DISCO) (Wing et al., J Child Psychol Psychiatry 43:307–325, 2002). The most PDA-specific subset of relevant DISCO items was selected, based on low endorsement in general across a sample of 153 individuals assessed for possible ASD using the DISCO. Having selected 11 DISCO PDA items for the measure, a subset of individuals with a high number of these features was identified (*N* = 27). Consistent with Newson’s descriptions, this high scoring group was characterised by lack of co-operation, use of apparently manipulative behaviour, socially shocking behaviour, difficulties with other people, anxiety and sudden behavioural changes from loving to aggression. All but one case met criteria for an ASD. This study brings the field a step closer to a clinician-rated measure of PDA features and highlights the need for further elucidation of the PDA phenotype.

**Electronic supplementary material:**

The online version of this article (doi:10.1007/s00787-015-0740-2) contains supplementary material, which is available to authorized users.

## Introduction

Pathological demand avoidance (PDA) is a term coined by Elizabeth Newson in the 1980s to describe children putatively within the spectrum of pervasive developmental disorders who exhibited an unusual pattern of behaviour [1]. Key characteristics included an obsessive resistance to everyday demands and a tendency to use a range of ‘socially manipulative’ strategies to subvert requests (e.g. distraction, targeted shocking behaviour, threats). A second feature was ‘surface sociability’—a superficial ability to manage social interaction, but with little evidence of a normal sense of social identity (e.g. believing themselves to be on a par with or superior to adults), and a lack of pride or shame, evident in socially shocking behaviour viewed as infantile or irksome by peers. A third feature was extreme impulsivity and lability of mood, apparently motivated by an obsessive need for control, and evident in domineering and volatile behaviour towards peers and even adults. A fourth characteristic was a tendency to appear comfortable in role play and pretending—often adopting borrowed roles when interacting with others (e.g. relating to peers in the manner of a teacher). Additional features were language delay, which was considered the result of passivity, obsessive behaviour often targeted at particular people or their characteristics, a passive early history and neurological involvement (e.g. delayed milestones, clumsiness, seizures or absences in a minority) [1].

Based on a review of clinical cases seen, Newson reported an equal gender ratio in PDA, in contrast with more typical presentations of autism spectrum disorder (ASD) [1, 2]. She also noted that those with this profile did not respond to educational and management approaches recommended for most individuals on the autism spectrum. Instead of structure and predictable routine, Newson suggested that children with PDA responded best to novelty, humour and flexibility. Development of one to one relationships with staff was reported to buffer demands, which could also be disguised using an ‘indirect’ approach [1, 3].

The concept of PDA has grown in popularity during the last decade, particularly in the UK where the description originated. Despite the absence of agreed diagnostic criteria for PDA, the limited research base and its lack of inclusion in the ICD-10 [4] or DSM-5 [5], clinicians are increasingly using the term to describe children who fit the profile. There has been debate with regard to the usefulness of PDA as a concept. However, the depth of interest in the topic is evidenced by annual oversubscribed conferences on PDA organised by the UK-based National Autistic Society (NAS) since 2011, and inclusion of guidelines on PDA and recommended teaching strategies as part of the national autism standards published by the UK-based Autism Education Trust [3]. Central to this enthusiasm is the sense that identifying PDA features in individuals within the autism spectrum may serve an important clinical function in providing tailored educational and support strategies [6]. The impetus for such work is strong given the very significant behavioural challenge that these individuals present [e.g. 1, 6, 4].

### Motivation for the present study

Despite interest and research into PDA increasing apace [e.g. 6, 7–11], as yet no clinician-rated instrument has been developed to quantify PDA features. One diagnostic tool that includes indicators of a number of features pertinent to PDA is the Diagnostic Interview for Social and Communication Disorders (DISCO) [12]. This semi-structured interview is widely used as an assessment tool for autism spectrum conditions and covers a wide range of behaviours associated with the phenotype. The original DISCO assessment included some items relevant to PDA and the instrument was later extended to include specific items capturing Newson’s description of PDA [12]. Although Wing and Gould’s draft 15-item PDA list comprising these items was never formally validated, it has been used, for example, to study the prevalence of PDA within a general population study of autism in the Faroe Islands [11]. Notably, Wing and Gould’s draft list covers some very distinctive characteristics of PDA (e.g. socially shocking behaviour), as well as less PDA-specific indicators (e.g. clumsiness, passive early history).

In the interim, a parent-report questionnaire measure (the Extreme Demand Avoidance Questionnaire (EDA-Q) [9]) has been developed. This measure was designed to assess the extent to which a child, based on parent-reported information, has a profile consistent with descriptions of PDA. The term ‘extreme’ rather than ‘pathological’ was used to avoid pejorative connotations. In the course of the validation study for the questionnaire, data were collected from a large sample of parents of children identified as having PDA by a clinician (*N* = 50) [7]. These data provide an initial source of information on how common particular traits and behaviours are in children reported to have PDA. The results are for the most part consistent with Newson’s early descriptions [1]. However, variability in endorsement rates across items suggest that certain features (e.g. avoiding demands) may be more central to the phenotype than other (e.g. passive early history). Data from the EDA-Q study also showed that whilst those children who are reported to have PDA scored significantly higher than comparison groups on the EDA-Q total score, a large proportion of those with autism plus behaviour problems (but who had not been identified as having PDA) also scored relatively high. This suggests that a number of the traits characteristic of PDA are not very specific to the PDA phenotype and may be relatively common across the autism and problem behaviour phenotypes.

The aim of the present study was to identify from within the DISCO items a set of indicators that are characteristic of and relatively specific to PDA, being uncommon in the autism spectrum in general. Scores on these items were then used to identify a PDA group from within a large sample of DISCO-assessed cases. Additional features captured in the DISCO assessment were compared between the PDA groups and the rest of the sample. This allowed us to explore behavioural overlap and differences between PDA and individuals being assessed for possible ASD without PDA features. The DISCO interview schedule consists of ratings of the severity of behaviours at the time of assessment (‘current’ ratings) and also of the most acute the behaviours had ever been (‘ever’ ratings). This made it possible to examine whether the severity of PDA features had declined in a proportion of the sample.

## Materials and methods

### Ethical approval

Ethical approval was obtained to analyse anonymised numerical scores for DISCO items and non-identifiable details (e.g. age, gender, diagnosis) from cases submitted by DISCO trainees, who completed a DISCO assessment of a case as part of their training at the Lorna Wing Centre between 2006 and 2010. Ethical approval was obtained from King’s College London (Psychiatry, Nursing and Midwifery Ethical Review Board).

### Analysis

#### Identification of DISCO PDA indicators

The first part of the study aimed to ascertain which PDA items might prove discriminating. This involved three stages. First, results from the EDA-Q study were used to identify ‘core features’ of PDA (high endorsement rates in the PDA group and high loadings on the first principal component [9]). Our criteria for an item to make the shortlist were an endorsement rate of 66 % or higher in the data from those reported to have PDA (*N* = 50) and an eigenvalue > 0.5 loading onto the first principal component. Online Resource 1 details a list of the 18 EDA-Q items meeting these criteria (for data on other EDA-Q items, see [9]; Tables S2 and S5). Notably, whilst items pertaining to what appear to be the core features of PDA are included (e.g. avoidance of demands and requests), items focusing on features such as ‘passive early history’ are omitted, due to lower eigenvalues and endorsement rates. Eighteen EDA-Q items met criteria as core features (Online Resource 1).

Second, DISCO items with similar content to those 18 EDA-Q items were identified. Table 1 lists the EDA-Q items and the closest corresponding DISCO items. Seventeen DISCO items appeared to provide a good match (Table 1), although given that the wording of these items is not identical, this could not be perfect.

**Table 1:** EDA-Q items that met inclusion criteria and the closest corresponding DISCO items that were identified

EDA-Q items (18 items)	Corresponding DISCO items (17 items)	DISCO variables
Obsessively resists and avoids ordinary demands	Lack of co-operation	LACKCOP
Has difficulty complying unless carefully presented		
Is driven by the need to be in charge	Using age peers as mechanical aids, bossy and domineering	CPEERAD*
Tells other children how to behave		
Finds everyday pressures intolerably stressful	Anxiety	ANXIETY
Mimics adult mannerisms and styles	Repetitive acting out roles	CTROL*
Shows little shame or embarrassment	Behaviour in public places	BEHAPUB
	Embarrassing remarks in public	REMARK
Good at getting around others	Apparently manipulative behaviour	MANBEH*
Unaware of differences between self and authority figures	Awareness of own identity	CIDENT*
Attempts to negotiate better terms with adults		
If pressurised to do things, may have a ‘meltdown’	Temper tantrums	TEMPER
Mood changes rapidly	Changeable mood	MOODCH
Knows what to do or say to upset specific people	Difficulties with other people	DIFPEOP
Blames or targets a particular person	Harassment of others	HARAS*
	Blaming other people	BLAME*
Denies behaviour, even when caught red-handed	Fantasising, lying, cheating, stealing	LYING*
Outrageous behaviour to get out of doing something	Socially shocking behaviour	SHOCK*
Extreme emotional responses to small events	Inappropriate sociability (rapid, inexplicable changes from loving to aggression)	CINAPP*
Social interaction has to be on his/her own terms	One-sided social approaches	CONESID

Items that were included in Wing and Gould’s draft 15-item PDA list are designated with an asterisk

The third stage used data from a sample of cases assessed using the DISCO for possible autism spectrum disorder (*N* = 153). These data were used to determine which of the 17 PDA-relevant DISCO items were *not* widely endorsed in general in an autism spectrum sample, since features typical of ASD in general are unlikely to be useful in identifying a meaningful subgroup. Ten of the DISCO PDA items had low endorsement rates (‘marked difficulties’ in less than 30 % of the total sample). Low endorsement suggested that these items might prove somewhat specific indicators of PDA. An additional item, ‘Lack of co-operation’, did not meet this low endorsement threshold (it was endorsed as ‘marked’ for 33 % of the sample), but was included due to its conceptual centrality—capturing resistance to demands. As can be seen from Table 5, this is the only item that corresponds directly to ‘Continues to resist ordinary demands’—an essential characteristic based on Newson’s descriptions.

It should be noted that the newly derived 11-item PDA DISCO measure included only 8 out of 15 items from Wing and Gould’s draft PDA list, which was recently used to examine the prevalence of PDA [11]. Items from Wing and Gould’s draft list that were omitted include five questions for which no parallel item existed in the EDA-Q. These items centred on traits not thought to be distinctive to PDA versus the rest of the spectrum (‘Clumsy in gross movements’, ‘Repetitive questioning’), or items that appeared too specific to reflect Newson’s description at a broader level (e.g. ‘Communicates through doll, puppet, toy animal etc.’, ‘Hands seem limp and weak for unwelcome tasks’), plus the item ‘Obsessed with a person, real or fictional’. One item that did have a parallel in the EDA-Q was excluded—‘Unusually quiet and passive in infancy’. The parallel EDA-Q item did not meet the cutoffs used here to reduce the list (endorsement >66 % and loading of 0.5 onto the first eigenvariate). One final item (‘Blames others for own misdeeds’) had a parallel in the EDA-Q and met the EDA-Q criteria, but the relevant DISCO item did not meet the low-frequency criterion for items in this sample. Differential endorsement for omitted items from Wing and Gould’s draft list is considered in detail in “Results”.

The second part of the analysis used the newly derived 11-item DISCO PDA measure to identify cases with high levels of PDA features from within the sample. Cutoff scores that required endorsement of a high proportion of the 11 items were used (see “Materials and methods” for details on how the cutoffs were derived). These cutoffs could not be validated against case report information or independent ‘diagnosis’ of PDA, since these were not available. However, establishing putative cutoffs made it possible to examine the profile that those with high levels of PDA features showed across other traits, to explore whether the subgroup was distinct in terms of aspects independent of the PDA item selection criteria. The quality of social interaction, social communication, social imagination and rigid and repetitive pattern of activities, maladaptive behaviours and emotional symptoms were compared across the groups that were high versus low on PDA features, to explore the degree of phenotypic overlap.

### Measures

The DISCO is a semi-structured interview administered by a clinician [12]. The clinician rates each item on the basis of information reported to them by the parent, the person being assessed or from other sources. For most DISCO items, two ratings are made, one indicating severity of the target behaviour at the present time (termed the ‘current’ rating) and the other reflecting the most severe the target behaviour has ever been (the ‘ever’ rating). Lower scores in the DISCO indicate more severe behaviour. The DISCO coding rules demand that ‘ever’ ratings are always lower or equal to the current ratings. Certain summary items are scored differently, with separate ratings for past behaviour and current behaviour. The analyses presented here focus on ‘current’ ratings, except when specifically indicated in the text.

#### Characteristics of the sample of cases for whom DISCO data were available

In the present study, the assessment of cases using the DISCO had been undertaken by clinicians who participated in the DISCO training courses held at the Lorna Wing Centre. Clinicians were from a range of disciplines and specialities (e.g. paediatrics, clinical psychology, speech and language therapy, nursing, psychiatry, educational psychology). Cases were included if the clinician’s assessment was deemed to have met satisfactory standards and the case reported was of an individual aged 5 years or older. A total of 153 cases were included in the sample. Course participants had been encouraged to choose a complex or puzzling case that would challenge them to develop their assessment skills. As such, the sample of cases constitutes a mixed clinical sample, possibly over-representing unusual cases.

### Participants

The sample ranged in age from 5 to 53 years, mean = 19.0 years. There were 77 adults (18–53 years, mean age = 27.5 years) and 76 children (5–17 years, mean age = 10.3 years). Table 2 details gender cross-tabulated with clinician-reported ability level. As part of the DISCO training, course participants were encouraged to assess their cases against ICD-10 criteria. If criteria for neither childhood autism nor Asperger syndrome were met, course participants were encouraged to assess against Gillberg’s Asperger criteria [13, 14]. Of the cases included in the sample, 94 received a diagnosis of autism or ASD as a result of the DISCO assessment, 28 received a diagnosis of Asperger syndrome and 6 did not receive a diagnosis of ASD. Ultimately, the choice of the diagnostic term was with clinicians, and therefore the use of terms outside of the ICD-10, but in common usage (e.g. ASD), was also reported.

**Table 2:** Cross-tabulation of gender and clinician-rated ability level in the sample of cases assessed using the DISCO

Gender (*N*)	Severe–moderate LD (%)	Mild LD–borderline (%)	Normal ability range (%)	Missing IQ (%)
Male (108)	19 (17)	35 (32)	46 (43)	8 (7)
Female (45)	10 (22)	17 (38)	13 (29)	5 (11)

Percentages indicate the proportion of individuals within each gender who were rated as having each ability level

For the remaining 25 cases, information about eventual diagnosis was not provided. This was because for some clinicians attending the DISCO training, diagnoses were routinely made by a team, and as such they would not have made a decision alone and submitted this as part of their assessment. Therefore, missing diagnostic information does not necessarily reflect diagnostic ambiguity. Ratings pertaining to meeting or not meeting criteria for autism spectrum disorder on the basis of summary items for quality of social interaction, social communication, social imagination and rigid and repetitive pattern of activities indicated that 150 of the sample were rated as currently exhibiting social interaction impairments characteristic of an ASD; 152 showed current impairments in social communication; 152 met criteria for social imagination; and 148 met criteria on quality of activities (i.e. rigid and repetitive behaviour). Of the 153 cases, 59 had additional diagnoses besides ASD. Fifteen had received a diagnosis of ADHD, 14 had psychosis/bipolar disorder/schizophrenia, 12 had epilepsy, 9 had depression/anxiety/emotional problems, 8 had conduct disorder/aggressive or challenging behaviour, 6 had genetic disorders, 4 had obsessive compulsive disorder, 2 had substance abuse problems, 1 had Tourette’s and 1 had received a diagnosis of PDA.

## Results

### Endorsement of PDA-relevant items across the sample

Endorsement of PDA-relevant DISCO items was examined across the 153 cases assessed using the DISCO, to identify which PDA-relevant items might be sufficiently unusual to be discriminating. Table 3 lists the items and their endorsement rates for ‘marked difficulties’, ‘minor difficulties’ and ‘no difficulties’ across the whole sample (*N* = 153). Using a cutoff of 30 % or less of the sample reported to have ‘marked’ difficulties resulted in the identification of ten items. One additional item, ‘Lack of co-operation’, was also included due to its centrality to descriptions of PDA. Details of the final list of PDA indicators are in boldtype in Table 3. Full descriptions of the DISCO interview probes and scoring criteria for these 11 items are provided in Online Resource 2.

**Table 3:** Endorsement frequencies for DISCO items identified in Table 2

Item description	% Score = 0 (marked)	% Score = 1 (minor)	% Score = 2 (unaffected)	% Missing/un-rateable
Anxiety	52.9	25.5	21.6	0
One-sided social approaches	44.4	34	7.8	13.7
Temper tantrums	35.3	30.7	33.3	0.7
Changeable mood	34	27.5	38.6	0
**Lack of co-operation**	**33.3**	**34**	**32**	**0.7**
Blaming other people*	33.3	18.3	45.8	2.6
Embarrassing remarks in public	30.7	28.1	33.3	7.8
Apparently manipulative behaviour*	**27.5**	**24.2**	**47.7**	**0.7**
Awareness of own identity*	**26.8**	**24.8**	**47.1**	**1.3**
**Behaviour in public places**	**21.6**	**29.4**	**49**	**0**
**Difficulties with other people**	**16.3**	**14.4**	**67.3**	**2**
Repetitive acting out roles*	**15**	**11.8**	**20.3**	**52.9**
Fantasising, lying, cheating, stealing*	**13.7**	**17**	**67.3**	**2**
Inappropriate sociability (rapid, inexplicable changes from loving to aggression)*	**13.1**	**18.3**	**46.4**	**22.2**
Using age peers as mechanical aids, bossy and domineering*	**7.8**	**10.5**	**80.4**	**1.3**
Socially shocking behaviour*	**7.8**	**7.2**	**84.3**	**0.7**
**Harassment of others**	**7.2**	**10.5**	**80.4**	**2**

Items that were included in Wing and Gould’s draft 15-item PDA list are designated with an asterisk. Items are sorted from most to least commonly rated as ‘marked difficulties’ within the sample (*N* = 153). The 11 DISCO PDA items deemed most useful in identifying PDA are shown in bold. Two items included in this list had specific scoring rules. ‘Repetitive acting out roles’ was only scored in the clear presence of some degree of apparent imaginative activities (rated across two separate DISCO items), and was un-rateable for over 50 % of the sample. ‘Inappropriate sociability (rapid, inexplicable changes from loving to aggression)’ was only rateable in the presence of interactions with peers, coded in a separate item

### Distribution of total scores on the 11-item DISCO PDA measure

**Fig. 1:**
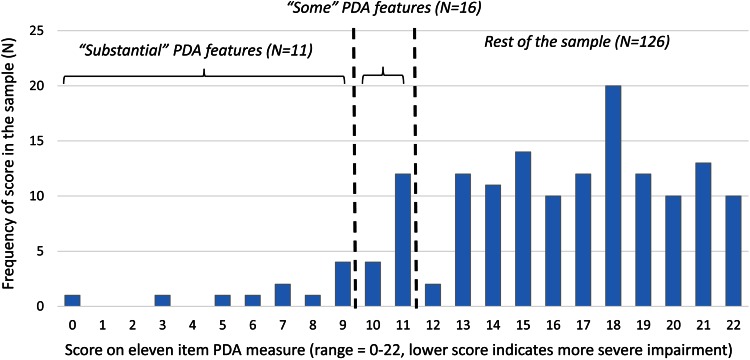
Distribution of total scores on the 11-item DISCO PDA measure (possible range of scores: 0–22; the lower the score, the more severe is the impairment)

Total scores on the 11-item DISCO PDA measure were calculated for all participants, by taking the mean score across the items and multiplying by 11. There was a minimum requirement of at least six codeable items to allow a total score to be calculated. This scoring method means that scores are not affected if, for some cases, certain items cannot be coded. Gender comparisons indicated the absence of significant group differences in the total score for the 11-item DISCO PDA measure (*t* (151) = 0.42, *p* > 0.1; mean for males = 15.75, mean for females = 15.43). There was also no significant relationship between age and total score (*r* = 0.12, *p* > 0.1), or clinician-reported ability and total score (*r* = 0.08, *p* > 0.1). Figure 1 illustrates the distribution of total scores on the DISCO PDA measure (possible range of scores: 0–22, with lower scores indicating more severe difficulties).

Identification of a cutoff is somewhat arbitrary, since scores followed a continuous (albeit skewed) distribution. However, we wanted to select a cutoff that would distinguish cases with a large number of PDA characteristics to enable further exploration of their profile on other indicators. A score of nine or below was selected to denote the most strikingly affected cases in this sample (described as having *“substantial”* PDA features). For such a score to be achieved, at least 2 of the 11 PDA DISCO items were endorsed as ‘marked’ and the remainder as ‘minor’, or a higher proportion as ‘marked’, with up to four features absent for any individual meriting this score. 11 out of 153 (7.2 %) cases met the threshold for *“substantial”* PDA features.

A total score higher than 9, but less than or equal to 11 could be said to identify individuals who have *“some”* PDA features. All characteristics on the list could be endorsed as ‘minor’ with none ‘marked’ or absent, or a higher proportion as ‘marked’ and up to five features absent, to achieve this score. Sixteen out of 153 (10.5 %) cases met the criteria for “*some”* PDA features.

It should be noted that these thresholds were identified for the purpose of the present exploratory analyses. To generate diagnostic cutoffs, it would be necessary to validate possible cutoffs against case report information and clinical judgements. Furthermore, given that nothing is known about the population representativeness (or lack thereof) of this sample, these figures cannot tell us about the incidence of extreme PDA features. It is, however, of note that achieving these scores within an already clinically complex sample is likely to denote PDA scores in an abnormal range within the general population, given that typical screening cutoffs for clinical features tend to identify cases in the top 5–10 % of the general population [e.g. 15].

### Characteristics of participants with “substantial” or “some” PDA features

Of the 11 participants in the sample with *“substantial”* PDA features, there were 8 males and 3 females (mean age = 15.7 years, range = 6–27 years). All seven participants under the age of 18 years were male (mean age = 9.6 years, range 6–13 years), while three of the four adult participants (mean age 24.8 years, range = 19–27 years) were female. Of the 16 participants in the sample with *“some”* PDA features, 10 were male and 6 female, with mean age 15.7 years (range = 6–41 years). Seven out of the 11 child participants (mean age 11.2 years, range = 6–17 years) and 3 out of the 5 adult participants (mean 25.6 years, range = 20–41 years) were male. Cross tabulation of sex by ability level for these groups is reported in Table 4.

**Table 4:** Cross-tabulation of gender and clinician-rated ability level in the two PDA groups

	Severe–moderate LD	Mild LD–borderline	Normal ability range	Missing ability information	Total
Substantial PDA features group					
Male	2	1	5	0	8
Female	0	3	0	0	3
Total	2	4	5	0	11
Some PDA features group					
Male	2	3	4	1	10
Female	1	4	1	0	6
Total	3	7	5	1	16

### Percentage endorsement of PDA measure items in the “substantial” and “some” PDA subgroups

**Fig. 2:**
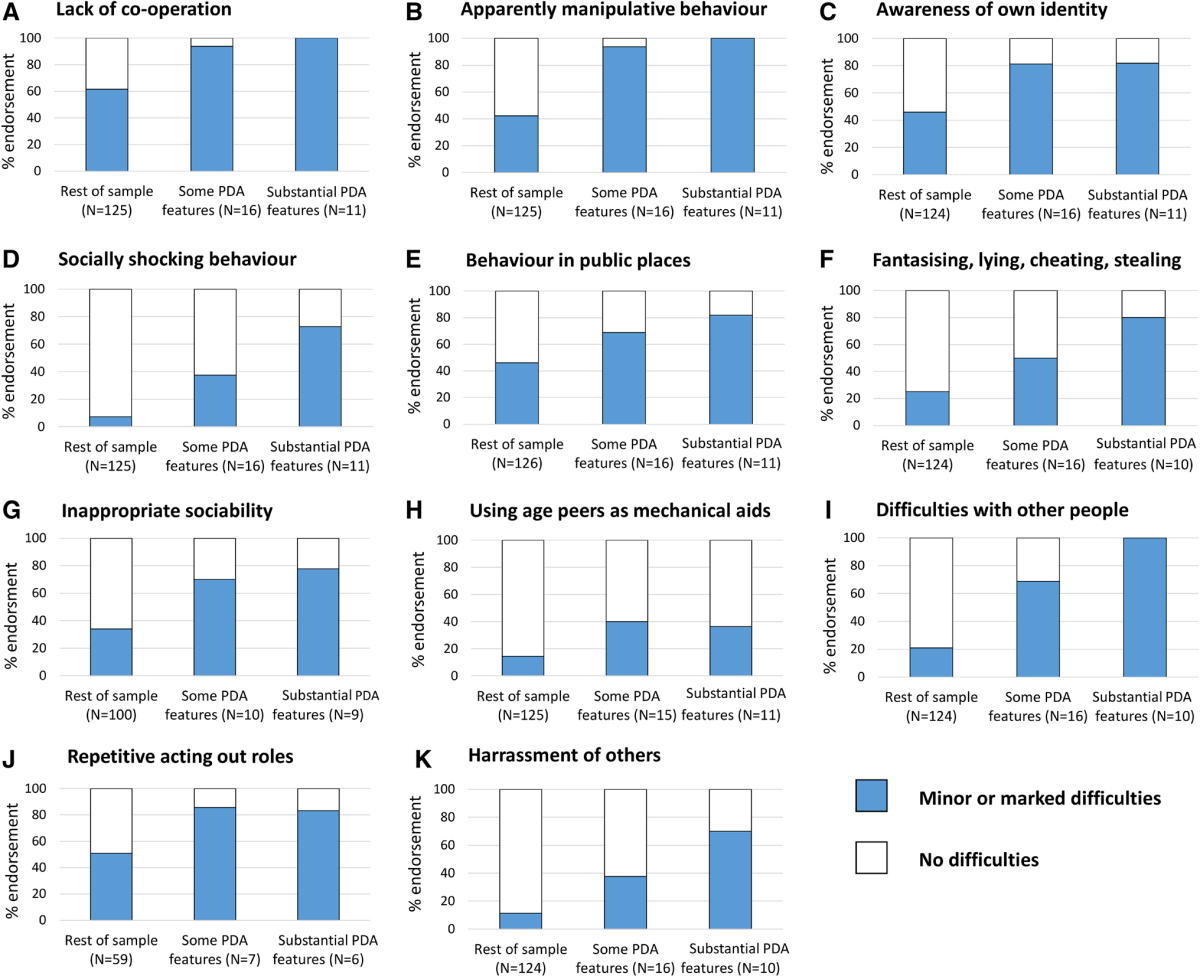
Percentage endorsement rates for items from the DISCO PDA measure stratified by group (*“substantial”* PDA features, *“some”* PDA features and the rest of the sample). *N*s reflect the number of codeable data points in each group for each item

Percentage endorsement of PDA-relevant items in those with *“substantial”* PDA features and *“some”* PDA features (referred to hereafter as ‘PDA groups’), as well as the remainder of the sample, is presented in Fig. 2. These results indicate that, for most of the items, there was relatively high endorsement in the PDA groups—all the items seem to be ‘pulling their weight’. Fisher’s exact test was used to compare differential endorsement for the PDA groups versus the rest of the sample. For six items (‘Lack of co-operation’; ‘Apparently manipulative behaviour’; ‘Awareness of own identity’; ‘Difficulties with other people’; ‘Harassment of others’; ‘Fantasising, lying, cheating, stealing’; ‘Socially shocking behaviour’), endorsement was significantly higher in PDA groups (*p* < 0.001, Fisher’s exact test, one sided, pooling scores for marked and minor difficulties). For ‘Behaviour in public places’ and ‘Using age peers as mechanical aids, bossy and domineering’, endorsement was significantly higher in the PDA groups (*p* < 0.01), and for ‘Inappropriate sociability (rapid, inexplicable changes from loving to aggression)’, at *p* < 0.05. For ‘Repetitive acting out roles’, increased endorsement in the PDA groups failed to reach the nominal significance threshold (*p* = 0.06). Because of the coding rules that required indication of some imaginative activities for this item to be coded, the sample size for this item was substantially lower than for the other items (*N* = 74).

Across the whole sample, the alpha for the 11-item DISCO PDA measure was 0.71. Item-total correlations are reported in Online Resource 3. All were at or around 0.30, with the exception of ‘Using age peers as mechanical aids, bossy and domineering’, which was 0.10.

### Change from ‘current’ to ‘ever’ ratings

As noted in “Materials and methods”, ratings for each item are made for ‘current’ and ‘ever’ patterns of difficulty, with the latter indicating the most severe the behaviour has ever been (which could reflect the present or past level). Data were examined to establish what proportion of cases had ‘ever’ ratings that placed them in either the *“substantial”* or *“some”* PDA features groups, but ‘current’ ratings placed them in a less severe group (i.e. they have moved from *“substantial”* to *“some”* PDA features, or to no longer meeting either threshold). This made it possible to gauge what proportion of the sample may have experienced a reduction in the severity of their PDA features over time.

**Fig. 3:**
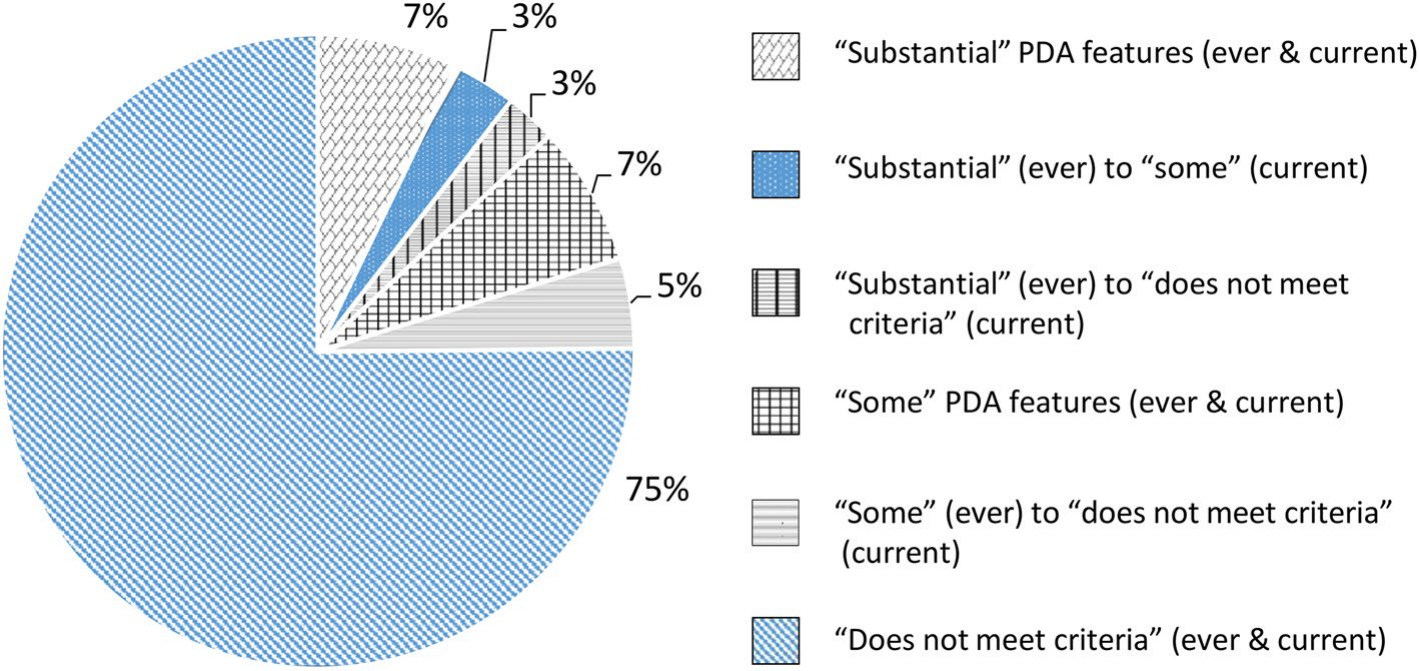
The proportion of the sample meeting thresholds “substantial” or “some” PDA features for both “current” and “ever” ratings in the sample. All groups span a range of ages: *“substantial”* (ever & current): 6–27 years, mean = 15.1 years; *“substantial”* (ever) to *“some”* (current): 7.6–14 years, mean = 19.9 years; *“substantial”* (ever) to *“does not meet criteria”* (current): 5.1–23 years, mean = 17 years, *“some”* (ever and current): 6–24 years, mean = 13.8 years, *“some”* (ever) to *“does not meet criteria”* (current): 7.5–27 years, mean = 20.0 years, *“does not meet criteria”* (ever and current): 5–53 years, mean = 19.7 years

A total of 115 participants (75 %) did not meet the criteria for PDA features on either current ratings or ever ratings. On ‘ever’ ratings, 18 participants (11.8 %) had *“some”* PDA features and 20 (13 %) had *“substantial”* PDA features. Out of the 20 who had ever had *“substantial”* PDA features, 11 participants still had *“substantial”* PDA features, 5 had *“some”* PDA features and 4 did not currently meet the criteria. Out of the 18 participants who had ever met the criteria for *“some”* PDA features, 11 were still meeting this threshold and the remaining 7 no longer met the criteria. These results are depicted in Fig. 3.

Whilst these results do suggest an improvement in the severity of PDA features over time in some participants, it is possible that for other participants (i.e. those who met the same thresholds on both ‘current’ and ‘ever’ ratings) their behaviour had in fact got worse over time. This would not be detectable using the DISCO coding, because for behaviour currently ‘at its worst’, current and ever ratings would be equal.

To examine whether ‘improvement’ in symptoms was age linked, an independent samples *t* test was conducted to examine whether there were significant differences in age between cases for whom ‘current’ and ‘ever’ ratings both indicated the presence of either *“some”* or *“substantial”* PDA features (*N* = 22, mean age = 14.5 years, range 6–27 years), and cases for whom ratings of ‘current’ behaviour indicated a reduction in severity of features compared to ‘ever’ scores (*N* = 16, mean age 19.2 years, range 5–41 years). The difference in mean age approached significance (*t* (36) = 1.85, *p* = 0.072, Cohen’s *d* = 0.61).

### Profiles of participants with PDA features on other DISCO indicators

This section provides further information about the characteristics of groups currently exhibiting *“substantial”* or *“some”* PDA features, in terms of endorsement of other DISCO items. Fisher’s exact test was used to formally compare endorsement of items across the two PDA groups combined (*N* = 27) versus the rest of the sample (*N* = 126) pooling ‘marked’ and ‘minor’ ratings. Firstly, endorsement rates for those items that were included in Wing and Gould’s draft 15-item PDA list, which did not meet criteria for inclusion in our 11-item DISCO PDA measure, were examined. ‘Clumsiness’ was the only item included in Wing and Gould’s draft list, but not in ours that did show significantly higher endorsement across the PDA groups (*p* = 0.02, Fisher’s exact test, two sided), reported in 73 % of the *“substantial”* PDA features group, 81 % of the “*some”* PDA features group and 53 % of the rest of the sample. Other items showed similar endorsement rates across groups (*p* > 0.1, Fisher’s exact test). For ‘unusually quiet and passive in infancy’, endorsement was 55 % for *“substantial”* and 38 % for *“some”* PDA features, and 40 % in the rest of the sample. For ‘uses a doll or toy to communicate’, these figures were 9, 6 and 6 %, respectively. For ‘repetitive questioning’, they were 55, 88 and 56 %, and for ‘hands limp and weak for unwelcome tasks’, they were 9, 19 and 17 %. For ‘obsession with a person’, they were 36, 25 and 32 %. The pattern of results was very similar for ‘ever’ ratings of these behaviours.

Profiles were compared for “*substantial”* and *“some”* PDA features groups combined, versus the rest of the sample, on ratings of the quality of social interaction, social communication, social imagination and rigid and repetitive pattern of activities (Online Resource 4). In addition, comparisons were made for a number of other DISCO items, included in the analysis due to their possible relevance to PDA (Online Resource 5). These analyses were not corrected for multiple comparisons and the results can only be considered exploratory.

The results indicate that those with high levels of PDA features and the rest of the sample appear to share similar qualitative impairments in terms of social interaction, social imagination and pretend play, and rigid and repetitive behaviours and activities (Online Resource 4). For ‘Quality of Social communication: current behaviour’, the results showed a tendency for those in the PDA group to have less acute difficulties (Freeman–Halton approximation of Fisher’s exact test, two-sided, *p* = 0.044) [16], though this marginally significant result would not have survived correction for multiple comparisons and should be viewed with caution.

A number of other DISCO indicators were more frequently endorsed in those with *“substantial”* or *“some”* PDA features compared to the rest of the sample (Online Resource 5). These included 23 out of the 24 DISCO items that tap ‘socially maladaptive behaviours’. Many of these behaviours were alluded to in Newson’s work, such as physical aggression, laughing at others’ distress, lack of awareness of psychological barriers, difficult or objectionable personal habits, needing constant supervision and demanding attention from caregivers (all at *p* = 0.001 or *p* < 0.001). Anxiety and a number of other emotional symptoms were reported at high rates both in those with PDA features and across the rest of the sample.

## Discussion

The aim of the present study was to identify interview items from within the DISCO that would make it possible to distinguish cases with PDA features, avoiding items tapping behaviours that are relatively common across the autism spectrum. The first part of the study focused on identification of these items, informed by (a) existing results suggesting which features are commonly observed in individuals reported to have PDA [9] and (b) new data reported here from a sample of cases assessed using the DISCO, which indicated the features likely to be relatively unusual in the autism spectrum in general, and therefore potentially specific to PDA. This analysis resulted in the identification of 11 PDA-relevant DISCO items. Table 5 contains the features of PDA as outlined by Newson and colleagues [1] alongside a list of these items. As can be seen from the table, coverage for the features that appear most distinctive for PDA as compared to the non-PDA ASD population is good. However, less PDA-specific items relating to neurological involvement, passive early history and language delay are not represented.

**Table 5:** The main features of PDA outlined by Newson and colleagues [1] and the 11 DISCO PDA items deemed most useful in identifying PDA, organised to correspond with Newson’s criteria

Newson’s description	Relevant DISCO item description	DISCO item code
Continues to resist ordinary demands with strategies of avoidance that are essentially ‘socially manipulative’	Lack of co-operation	LACKCOP
	Apparently manipulative behaviour	MANBEH*
Surface sociability, but lack of sense of identity, pride or shame	Awareness of own identity	CIDENT*
	Socially shocking behaviour	SHOCK*
	Behaviour in public places	BEHAPUB
	Fantasising, lying, cheating, stealing	LYING*
Lability of mood, impulsive, led by need to control	Inappropriate sociability (rapid, inexplicable changes from loving to aggression)	CINAPP*
	Using age peers as mechanical aids, bossy and domineering	CPEERAD*
	Difficulties with other people	DIFPEOP
Comfortable in role play and pretending	Repetitive acting out roles	CTROL*
Obsessive behaviour (often social in nature)	Harassment of others	HARAS*
Neurological involvement	None included	
Passive early history	None included	
Language delay	None included	

Items that were included in Wing and Gould’s draft 15-item PDA list are designated with an asterisk. Full DISCO item descriptions are given in Online Resource 2

The second part of the study used the 11-item DISCO PDA measure to select cases with *“substantial”* PDA features or *“some”* PDA features from within the sample. These sub-groups, designated for the purposes of the present exploratory analyses, were then compared with the rest of the sample on a number of DISCO indicators. For the PDA DISCO items, very high endorsement (≥70 %) was observed in the substantial PDA features group, with good differential endorsement between PDA groups and the rest of the sample. This indicates that most of the PDA DISCO items were ‘pulling their weight’. Only one item (“using age peers as mechanical aids, bossy and domineering”) was much less endorsed in the PDA groups (though still significantly more so than in the rest of the sample), perhaps because the wording highlights ‘using peers as mechanical aids’, rather than a broad tendency to adopt domineering behaviour. Given that domineering behaviour was reportedly very common in PDA on the basis of the EDA-Q data, a change in the emphasis of this item could make it more sensitive to detecting these putative features of PDA.

Seven of the 15 DISCO items that had been included in a draft PDA list developed by Wing and Gould did not meet inclusion criteria for our measure. Six out of these seven items failed to show differential endorsement between the PDA groups (ascertained based on scores on our 11-item measure) and the rest of the sample. Wing and Gould’s draft list had used published descriptions by Newson of PDA features to generate an item pool. Notably, Newson’s descriptions were not specifically focused on the characteristics that can delineate PDA from the rest of the autism spectrum and were not ‘weighted’ in terms of which items were considered to be most central in the profile. The approach taken here to select items was aimed at exploring the possible differentiation of PDA and focusing on items that were most ubiquitous to the profile.

The final section of the analysis highlighted a number of additional items that appeared to differentiate PDA from the rest of the sample (Online Resource 5). These indicators included physical aggression, laughing at others’ distress, lack of awareness of psychological barriers, difficult or objectionable personal habits, needing constant supervision and demanding attention from caregivers. Many of these behaviours also featured in Newson’s original descriptions [1]. These findings suggest that future diagnostic formulations of PDA may need to incorporate these features.

Particularly striking in these results is the multiplicity of socially maladaptive behaviours with which individuals with PDA features present. This is consistent with the huge challenge in terms of behavioural management that individuals with PDA can pose [1, 6] and highlights the imperative for tailored interventions.

### Change in PDA features over time

The availability of ‘current’ and ‘ever’ ratings on the DISCO PDA indicators made it possible to identify a proportion of cases that appeared to have undergone some improvement in the severity of their difficulties. Figure 3 indicates that 44 % of the cases who had ever experienced *“substantial”* or *“some”* PDA features appear to have undergone an improvement and moved to a less severe grouping. Longitudinal studies are needed to fully interrogate change in severity of difficulties over time, because the DISCO scoring metric does not capture worsening difficulties. These may have occurred in those who appeared to remain at the same cutoff for their ‘current’ and ‘ever’ ratings (56 % of those who had ever experienced *“substantial”* or *“some”* PDA features). It would be of interest in future work to explore what factors promote the remission of some of the PDA features over time.

Across the sample, there was no effect of age on total scores for the DISCO PDA measure. Although a similar proportion of children and adults met the criteria in this study, more research using a population-representative cohort is needed to establish whether this reflects patterns across a community sample, or whether it reflects the fact that adults with this profile are more likely to come to clinical attention than adults with more typical ASD.

### The relationship between PDA and ASD

The analyses presented here highlight the overlap between PDA subgroups and the rest of the sample in terms of the nature and quality of difficulties in social interaction, social imagination, and rigid and repetitive pattern of activities (Online Resource 4). In contrast with the emphasis Newson placed on strong imaginative abilities in PDA, 6 out of the 24 in the PDA groups for whom ratings were available were rated as not showing pretend play, 2 had some learnt play, 3 engaged in pretend play that was copied, 5 engaged in creative but repetitive pretend play and 7 engaged in shared/pretend role play, but dominated/insisted on it being done in a particular way. Only one exhibited flexible/age-appropriate pretend play (Online Resource 4). These proportions do not differ significantly from those within the non-PDA group. These results could be due to clinicians using the DISCO having greater sensitivity to difficulties or abnormalities in pretend play. Alternatively, it could reflect differences in gender ratio in our PDA groups and Newson’s work. Indeed, reports have suggested a link between better imaginative abilities in females versus males on the spectrum [17, 18]. More research is needed to examine the purported links between PDA features, female gender and imaginative abilities. In particular, use of cognitive assessments to probe imagination would make it possible to measure aspects such as imaginative creativity, as opposed to repetitive engagement with fictional characters or roles, behaviour that is copied directly from others or even confabulated accounts of events. These latter three forms of ‘imagination’ may have contributed to the patterns of behaviour Newson described. Impairments in pretend play in PDA reported here provide further evidence for the overlap between PDA and ASD, and suggest that to understand PDA we must find out why a proportion of those with typical ASD features also exhibit extreme demand avoidance.

When Newson first described PDA, she conceived of it as a concept as separate from autism, but part of a set of ‘pervasive developmental disorders’. This was partly due to the apparent usefulness of novelty, humour and flexibility as strategies to encourage compliance—very different from the routine and predictability at the heart of ASD strategy [6]. These differences could suggest that individuals described as having PDA are less rigid than their non-PDA ASD counterparts.

However, these data suggest substantial levels of rigidity in the PDA groups. In terms of ratings of past behaviour, 7 out of 26 with PDA features for whom ratings were available were described as engaging in only repetitive activities, with 18 having some varied interests, but with prominent repetitive activities. Groups did not differ significantly from the non-PDA ASD section of the cohort for past and current behaviour. One interpretation of these observations is that in PDA, rigidity could centre on having control over one’s activities in the context of social interactions, as opposed to the temporal order of tasks or location of objects. Avoidance itself could be a manifestation of rigidity (e.g. an aversive response to the change in status from being in control to submitting to someone else’s will). More detailed analyses of the behaviour and responses of individuals with PDA are needed to examine this further.

All but one of those in the PDA groups met criteria for ASD on the basis of qualitative ratings made by clinicians. The one participant who did not was not rated as meeting ASD thresholds on any of the summary items. From these data, it is not possible to tell whether this reflects the absence of ASD features or subtle features that were missed in the assessment.

### Gender ratio in PDA

Whilst Newson and colleagues reported an even gender ratio in PDA [1], here, there were 18 males and 9 females in the PDA group, a similar gender ratio to the non-PDA cases in this sample. One possibility is that the items incorporated in our PDA measure might disproportionately focus on the more outwardly challenging, as opposed to passive, behaviours described in PDA. The latter have been reported to be more common in females with ASD [19]. Despite this, we found no significant differences between genders for scores on the 11-item DISCO PDA measure across this sample. Analyses in larger samples using case report and diagnostic information on PDA are needed to examine whether items tapping passive forms of demand avoidance (e.g. selective mutism) warrant inclusion in a PDA measure.

### Strengths and limitations

One of the strengths of the current study was that the data used were collected in 2010 or earlier: for the most part prior to the large peak in interest in PDA and the series of annual conferences on the topic held in the UK. As such, it is likely that clinicians were not particularly ‘on the lookout’ for PDA features in their cases. This meant that it was possible to get an honest and unbiased picture of the features of PDA in this sample.

Limitations of the present study include that the representativeness of the sample as a group undergoing assessments for social and communication disorders is unknown. As such, these results do not provide information about the prevalence of PDA features, or how they compare to a population cohort of those with autism. However, these data remain useful as a large sample of cases undergoing assessment for possible social and communication disorder.

Further limitations include the fact that cases not specifically suspected of social communication disorders were not included, and that none of the cases was comprehensively clinically reviewed by experienced clinicians for the purpose of making or refuting a clinical diagnosis of PDA. For a minority of participants, diagnostic information with respect to autism spectrum disorders was also unavailable. The cutoffs selected here were made pragmatically to ensure that a sufficient number of PDA features were present in the cases included in the PDA groups. However, these data do not provide information on the degree of day to day functional impairment these difficulties produced. As such, it is possible that a lower or a higher threshold may have been more appropriate for the identification of PDA features.

### PDA features in the rest of the autism spectrum

The data presented here highlight that a number of traditionally identified ‘PDA features’ are in fact quite common across the autism spectrum. These include lack of co-operation, changes in mood, anxiety, blaming others and making embarrassing remarks in public. Whilst these characteristics are present in Newson’s descriptions, they may reflect a much broader pattern of behaviours that are very typical of ASD and reflect poor social awareness, egocentricity, rigidity and social anxiety. In contrast, other features characteristic of PDA very clearly differentiated PDA-like individuals within the ASD group. These included (amongst others) apparently manipulative behaviour, difficulties with other people, harassment of others, fantasising, lying, cheating, stealing and socially shocking behaviour. As such, the issue of whether a more distinctive set of PDA items is helpful in designating a specific group of children who may have differential needs and prognosis to the wider ASD group warrants further investigation.

This study provides an important step towards refining the concept of PDA, highlighting the specific and striking characteristics of those who most resemble Newson’s descriptions. The imperative behind this work is the very significant behavioural challenge this sub-group present compared to most individuals with ASD [1, 6, 7]. Questions remain as to whether these individuals are on a continuum with those who display milder levels of these features who span the autism spectrum, or whether this sub-group have a different type of ‘social coding problem’ in addition to the difficulties that underpin their more typical autistic features.

Identifying sub-groups with particular patterns of behavioural features could have a number of important functions. First, studying individuals with a more homogenous symptom profile could increase the chance of identifying genetic influences and pathways to atypical development. Second, an accurate description of the individual’s behaviours and how these impact their day to day functioning is crucial to inform those involved in their care. Appropriate descriptions facilitate the development of targeted intervention approaches, and makes it possible to measure their success with regard to areas of difficulty that have most impact for the individual. For these reasons, we propose that collecting information on PDA features using the present items (or alternatives, [9]) will be a valuable addition to studying the course of development and the impact of interventions.

### Future directions

A number of important further questions remain with regard to using the DISCO to identify PDA. Firstly, studies are needed to validate appropriate cutoffs for the PDA measure. Better characterisation of the behavioural phenotype of PDA may motivate the incorporation of more of the DISCO items into a PDA measure. In addition, a number of further questions remain about PDA and its relationship with other profiles. Studies of PDA features in a large clinic sample of children with a range of backgrounds and developmental features beyond autism spectrum disorders are essential to expand our understanding of this profile. It may also be of interest to examine attachment patterns and the processes by which these may come about in children with PDA. Lastly, these data suggest that a subset of those individuals reported to have PDA features may experience some remission over time. It would be of great clinical interest to determine what factors may promote this.

In conclusion, this study provides a first step towards developing a measure to quantify PDA features using a standardised diagnostic interview, the DISCO. Extensive further work is needed to characterise the features of PDA in more detail and test the validity of this measure.

## Electronic supplementary material

Below is the link to the electronic supplementary material.

Supplementary material 1 (PDF 902 kb)
