# Supplementary material for: Identifying features of ‘pathological demand avoidance’ using the Diagnostic Interview for Social and Communication Disorders (DISCO)
Source: Eur Child Adolesc Psychiatry. 2015 Jul 30;25:407–19. doi: 10.1007/s00787-015-0740-2 (PMC4820467; doi:10.1007/s00787-015-0740-2)
Supplement: Supplementary file 1 — Supplementary material 1 (PDF 902 kb) [file 787_2015_740_MOESM1_ESM.pdf]

Online Resources for O'Nions, Gould, Christie, Gillberg Viding & Happé: Features of 'Pathological Demand Avoidance' identified using the Diagnostic Interview for Social and Communication Disorders ('DISCO').

Corresponding author affiliation: UCL and King's College London. e.o'nions.ucl.ac.uk

Online Resource 1: Items from O'Nions et al., 2014, JCPP, 55, 758-768 that loaded onto the first eigenvariate with eigenvalues greater than .55, and with endorsement in the PDA group <=66%.

EDA-Q item		*Eigenvalue (1st PC)*	*Endorsment % PDA iden.*
1	Obsessively resists and avoids ordinary demands	0.798	94
3	Is driven by the need to be in charge	0.792	90
4	*Finds everyday pressures intolerably stressful	0.657	80
5	Tells other children how to behave	0.793	88
6	Mimics adult mannerisms and styles	0.649	74
7	Has difficulty complying unless carefully presented	0.720	96
9	Shows little shame or embarrassment	0.559	72
11	Good at getting round others	0.739	82
12	*Unaware of differences between self and authority figures	0.720	84
13	If pressurised to do things, may have a 'meltdown'	0.768	94
15	Mood changes rapidly	0.747	92
16	Knows what to do or say to upset specific people	0.58	80
17	Blames or targets a particular person	0.678	78
18	Denies behaviour, even when caught red handed	0.628	76
21	Outrageous behaviour to get out of doing something	0.715	66
22	*Extreme emotional responses to small events	0.681	82
23	Social interaction has to be on his/her own terms	0.729	94
25	Attempts to negotiate better terms with adults.	0.552	84

Endorsement percentages indicate the proportion of parents who rated the item 'mostly true' or 'very true' for their child. Abbreviations: PC = principle component; PDA iden = group reported to have been clinically identified as having PDA (N=50).

Online Resource 2: Full descriptions of DISCO items included in the final 11-item list.

Item description
**Lack of co-operation (LACKCOP)**<br>Does A strongly resist attempts to make her/him join in, learn new things, or to change behaviour; screams, has temper tantrums, scratches, bites, kicks or passively resists, or uses distracting techniques?<br>0 Marked<br>1 Minor<br>2 No problem
**Apparently manipulative behaviour (MANBEH)**<br>Does A avoid demands by what appears to be socially manipulative strategies? (e.g. distracting the adult making demands, making an excuse such as "I have to do this first", runs away, hides, removes clothes, uses doll or puppet to make excuse. If all else fails, screams, hits, kicks in a panic).<br>0 Marked<br>1 Minor<br>2 No problem
**Awareness of own identity (CIDENT)**<br>Is A aware of the age or social group to which he/she belongs? For children ask if A realises he/she is a child. For older children and adults ask about identification with work mates, social class. Does A give adults due respect?<br>0 Marked lack of awareness<br>1 Minor<br>2 No problem
**Socially shocking behaviour (SHOCK)**<br>Does A shock other people by unexpected inappropriate actions for no apparent reason? (e.g. tearing up another person's work, pulling off someone's spectacles, taking pants down and urinating on floor, injuring someone else's pet animal).<br>0 Marked<br>1 Minor<br>2 No problem
**Behaviour in public places (BEHAPUB)**<br>How does A behave when taken into shops, restaurants, other enclosed public spaces? Is it possible for one parent alone to take A into such places?<br>0 Major problem with outings<br>1 Minor<br>2 No problem
**Fantasising, lying, cheating, stealing (LYING)**<br>Does A talk about fantasies as if real, or lie, or cheat, or steal?<br>0 Marked<br>1 Minor<br>2 No problem

Inappropriate sociability (rapid, inexplicable changes from loving to aggression) (CINAPP)

Does A at first sight appear to be sociable and friendly but can slip from loving to violent behaviour or vice versa for no apparent reason? May show both together, e.g. saying ‘I hate you’ in a sweet voice while hugging. May hug others too long and too hard.

0 Marked

1 Some problem

2 No problem

-8 No interaction

Using age peers as mechanical aids, bossy and domineering (CPEERAD)

Does A use age peers solely as aids in own activities, e.g. to collect materials, to assist in building some construction, to take a specified part in a scenario created by A?

0 Marked, frequent

1 Minor, occasional

2 No problem

Difficulties with other people (DIFPEOP)

Does A frequently tease, bully, refuse to take turns, make trouble?

0 Marked

1 Minor

2 No problem

Repetitive acting out roles (CTROL)

Does A act out the role of an object, animal, fictional person or real person so that A seems to become the acted role – it is not just pretence? Is this a major part of A’s play?

0 Marked

1 Minor

2 No problem

-8 Des not engage in role play.

Harassment of others (HARAS)

Does A harass other people? (e.g. writing threatening letters, making verbal threats, stalking, untrue accusations of sexual abuse)

0 Marked

1 Minor

2 No problem

Online Resource 3: Item total correlations for items in the 11 item measure.

	Corrected Item-Total Correlation (N=62)*	Corrected Item-Total Correlation (N=114)**
Lack of co-operation	.514	.559
Apparently manipulative behaviour	.506	.435
Awareness of own identity	.359	.299
Socially shocking behaviour	.442	.444
Behaviour in public places	.261	.305
Fantasising, lying, cheating, stealing	.328	.355
Inappropriate sociability (rapid, inexplicable changes from loving to aggression)	.340	.350
Using age peers as mechanical aids, bossy and domineering	.099	.134
Difficulties with other people	.481	.518
Repetitive acting out roles	.297	
Harassment of others	.338	.342

Note: *N=62 of 153 had full data on all of the items. N=114 had full data on all of the items if the role play item ('Repetitive acting out roles') was omitted.

Online Resource 4: Summary measures of social interaction, social communication, social imagination, and rigid and repetitive pattern of interests and behaviours.

A: Social interaction stratified by PDA group: ratings of past behaviour.

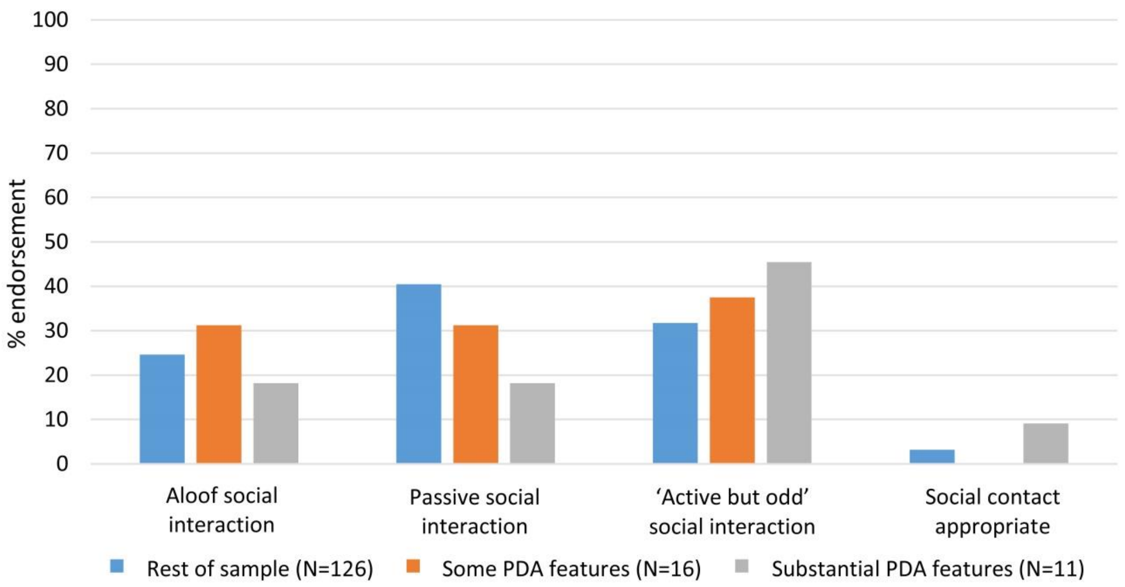

Ratings incorporated into the 'Aloof' interaction are: (1) 'Does not interact', (2) 'Interacts to obtain needs' and (3) 'Responds to physical contact'. For 'Passive' interaction, item is 'Does not initiate but responds to social contact'. For 'Active but odd' interaction, ratings are (1) 'Initiates contact but one sided' and (2) 'Stilted interaction'. There was no significant difference in ratings for the two PDA groups (N=27) vs. the rest of the sample (N=126) (Freeman-Halton extension of Fisher's exact test, two-sided, p=.202).

B: Social interaction stratified by PDA group: ratings of current behaviour.

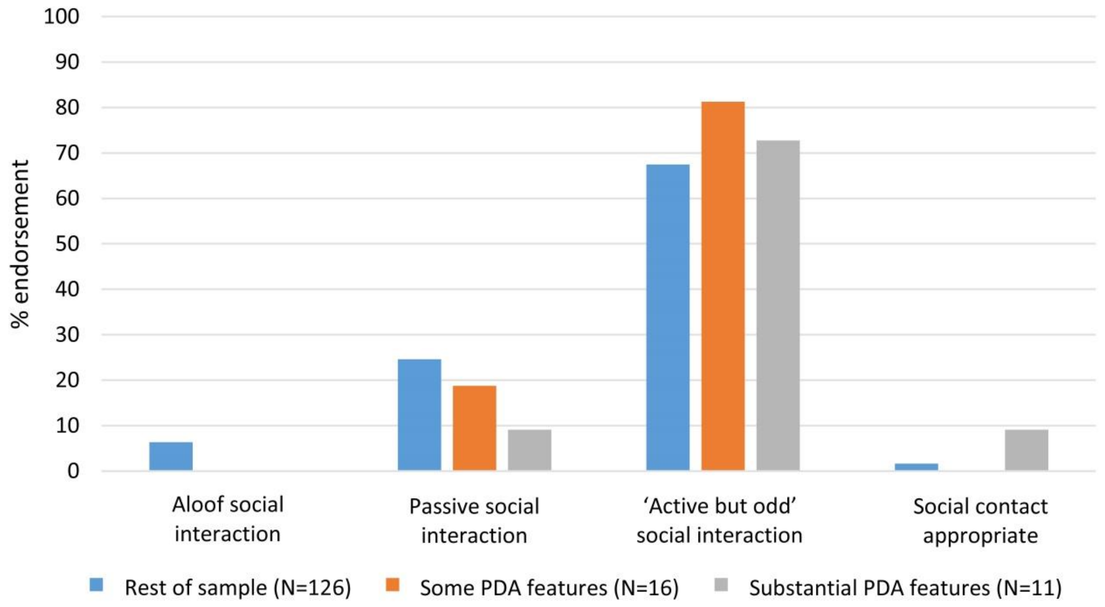

Ratings incorporated into the 'Aloof' interaction are: (1) 'Does not interact', (2) 'Interacts to obtain needs' and (3) 'Responds to physical contact'. For 'Passive' interaction, item is 'Does not initiate but responds to social contact'. For 'Active but odd' interaction, ratings are (1) 'Initiates contact but one sided' and (2) 'Stilted interaction'. There was no significant difference in ratings for the two PDA groups (N=27) vs. the rest of the sample (N=126) (Freeman-Halton extension of Fisher's exact test, two-sided, p=.104).

C: Social communication stratified by PDA group: ratings of past behaviour

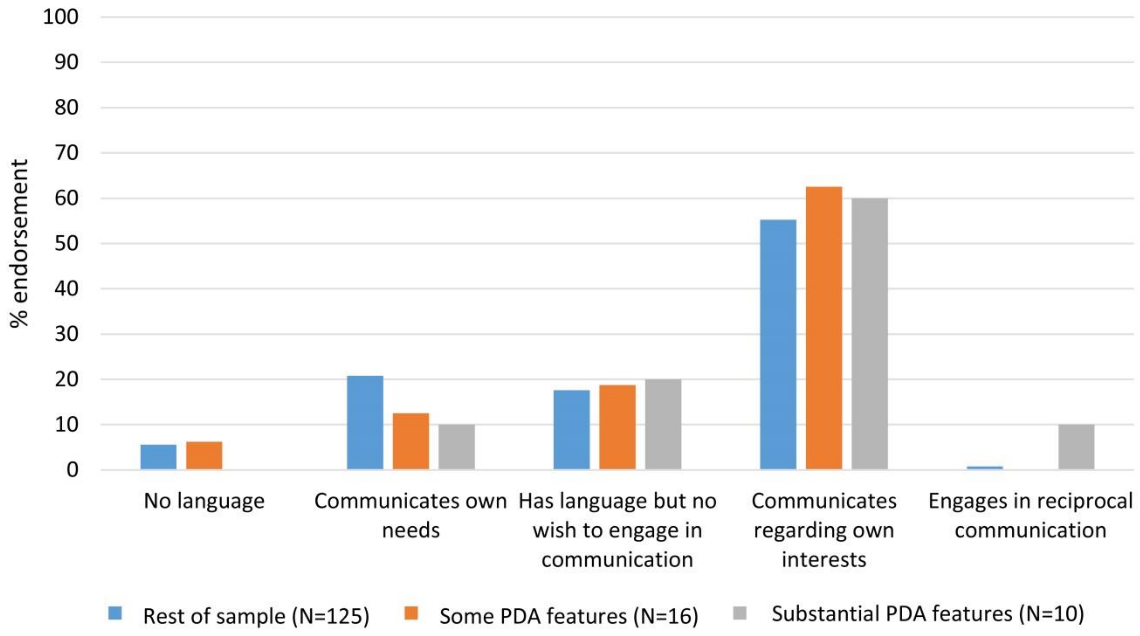

There was no significant difference in ratings for social communication: ratings of past behaviour between the two PDA groups combined (N=26) vs. the rest of the sample (N=125) for (Freeman-Halton extension of Fisher's exact test, two-sided, p=.616).

D: Social communication stratified by PDA group: ratings of current behaviour

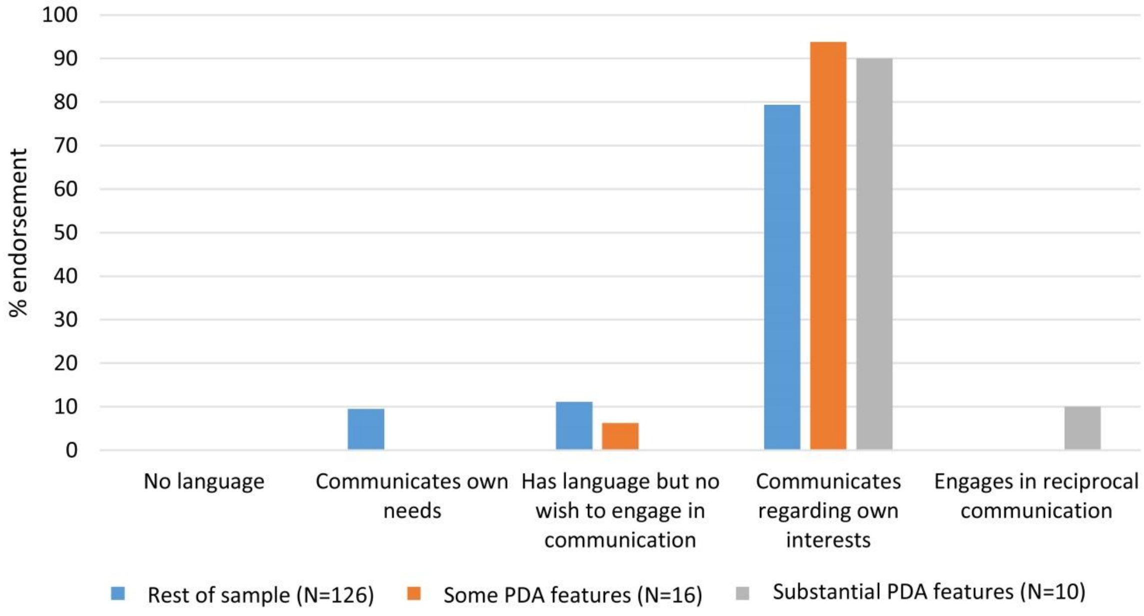

The difference in ratings for social communication: ratings of current behaviour for the two PDA groups combined (N=26) vs. the rest of the sample (N=126) reached the threshold for nominal significance (Freeman-Halton extension of Fisher's exact test, two-sided, p=.044). However, this result would not survive correction for multiple comparisons.

E: Social imagination/ pretend play stratified by PDA group: ratings of past behaviour

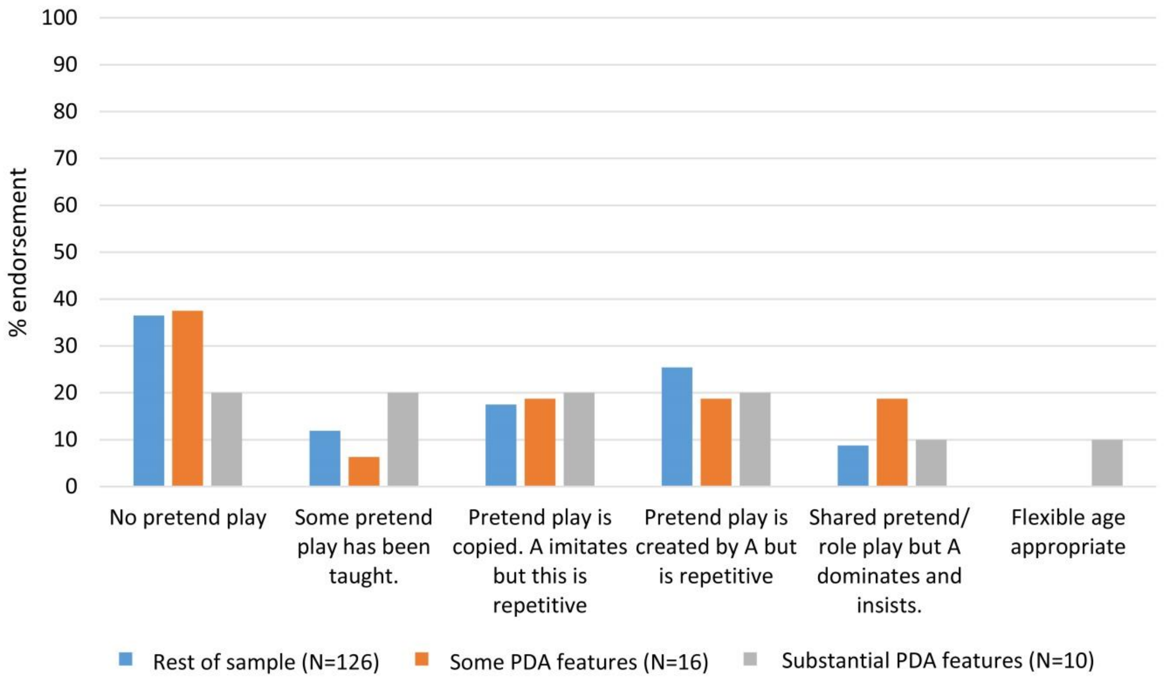

There was no significant difference in ratings of Social imagination/ pretend play: ratings of past behaviour for the two PDA groups (N=26) vs. the rest of the sample (N=126) (Freeman-Halton extension of Fisher's exact test, two-sided, p=.383).

F: Social imagination/ pretend play stratified by PDA group: ratings of current behaviour

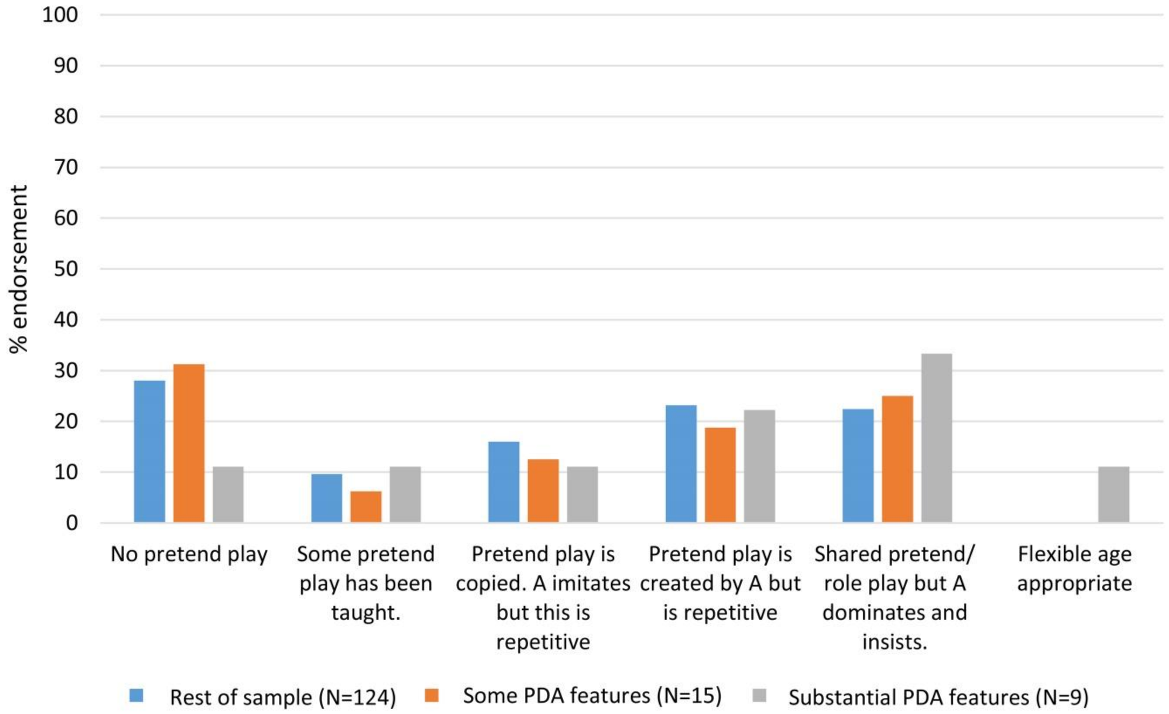

There was no significant difference in ratings of Social imagination/ pretend play: ratings of current behaviour for the two PDA groups (N=24) vs. the rest of the sample (N=124) (Freeman-Halton extension of Fisher's exact test, two-sided, p=.372).

G: Rigid and repetitive pattern of activities stratified by PDA group: ratings of past behaviour

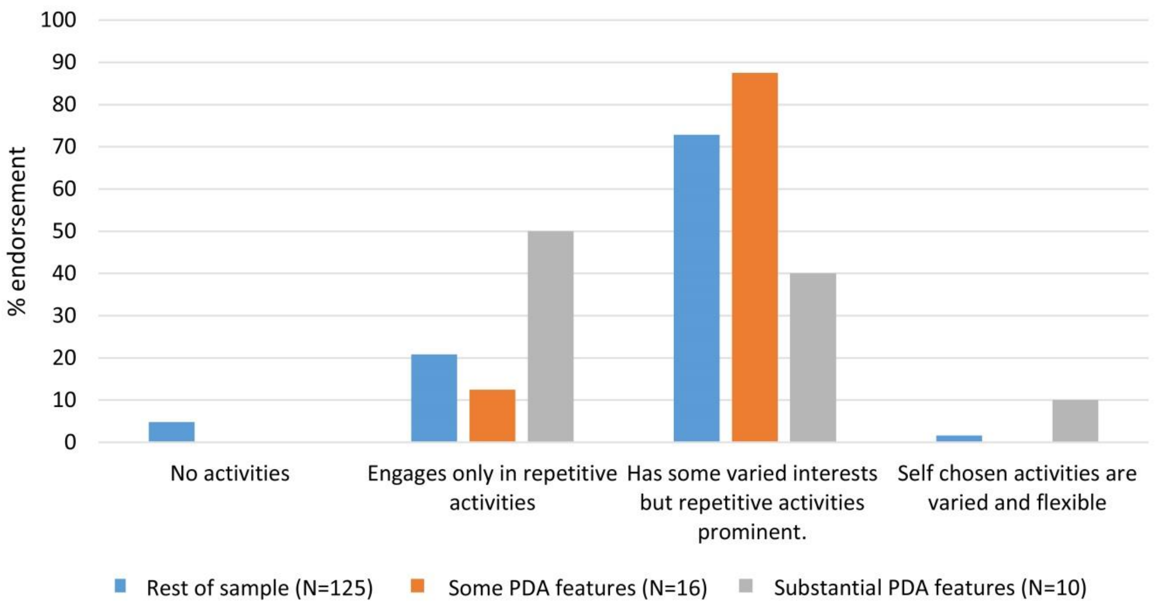

There was no significant difference in ratings of rigid and repetitive pattern of activities: past behaviour for the two PDA groups combined (N=26) vs. the rest of the sample (N=125) (Freeman-Halton extension of Fisher's exact test, two-sided, p=.455).

H: Rigid and repetitive pattern of activities stratified by PDA group: ratings of current behaviour

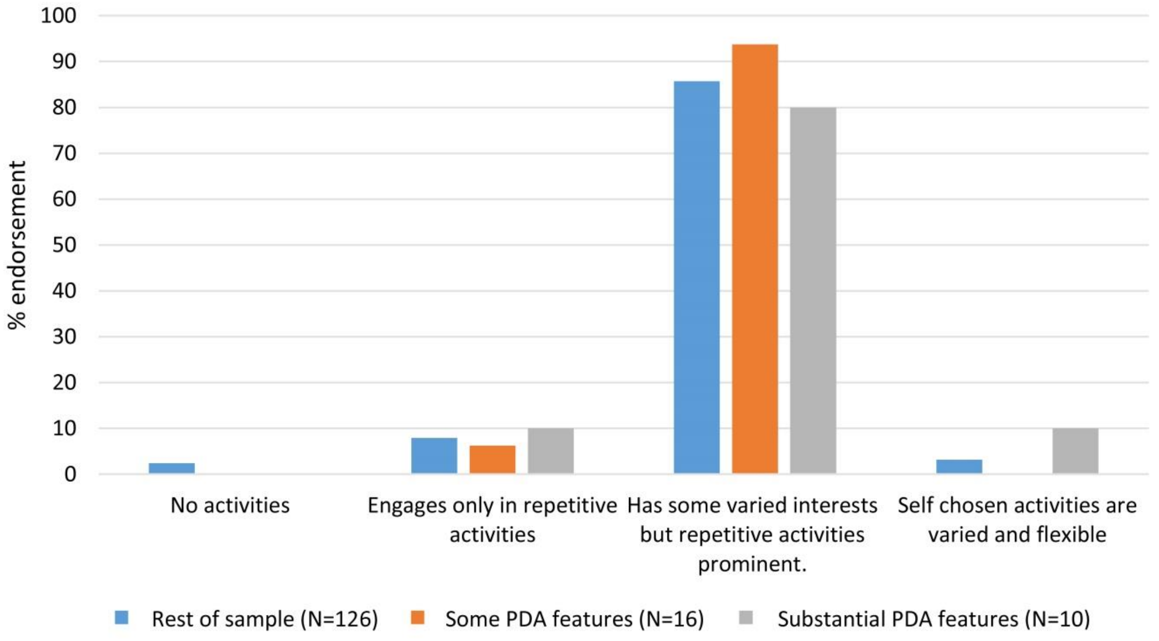

There was no significant difference in ratings of rigid and repetitive pattern of activities: current behaviour for the two PDA groups combined (N=26) vs. the rest of the sample (N=126) (Freeman-Halton extension of Fisher's exact test, two-sided, p=1.0).

Online Resource 5: Endorsement of DISCO items selected for potential relevance to PDA across the PDA groups and the rest of the sample

		% MINOR OR MARKED			PDA vs. Rest of the sample (Fisher's exact test, 2 sided)
		Rest of sample	Some PDA features	Marked PDA features	
PART 3 - SECTION XII - SOCIAL INTERRACTION - WITH ADULTS					
**Awareness of own identity**	**N=151**	**46**	**81**	**82**	**p < .001**
Does not seek comfort when in pain or distress	N=151	62	56	64	p > .1
Does not give comfort to others	N=152	81	88	91	p > .1
One sided social approaches	N=132	91	93	90	p > .1
Lacks awareness of others' feelings	N=152	91	100	91	p > .1
**Laughs at others' distress**	**N=153**	**41**	**63**	**100**	**p < .001**
Fascination with violence	N=152	23	19	45	p > .1
Lacks reaction to others' happiness	N=140	81	79	90	p > .1
Limited sharing of interests and enjoyment	N=153	83	100	82	p > .1
Avoidance of age peers	N=153	50	69	36	p > .1
PART 3 - SECTION XIII - SOCIAL INTERACTION - WITH AGE PEERS					
Lack of interaction with age peers	N=153	71	50	55	p = .072
Peers are not referred companions	N=124	66	77	56	p > .1
Quality of interaction impaired	N=117	94	100	100	p > .1
**Inappropriate sociability (rapid, inexplicable changes from loving to aggression)**	**N=119**	**34**	**70**	**78**	**p = .021**
Lacks emotional response to age peers	N=123	82	93	100	p > .1
Lacks conventions of peer interaction	N=101	85	100	100	p > .1
**Using age peers as mechanical aids, bossy and domineering**	**N=151**	**14**	**40**	**36**	**p = .011**
Lacks friendship with age peers	N=133	60	79	82	p > .1
Quality of friendship impaired	N=47	67	100	100	p = .074
**Bullying and teasing by age peers**	**N=132**	**57**	**73**	**100**	**p = .040**

Items marked in bold reached the nominal significance threshold for increased endorsement in the PDA groups vs. the rest of the sample (Fisher's exact test, two sided).

		% MINOR OR MARKED			PDA vs. Rest of the sample (Fisher's exact test, 2 sided)
		Rest of sample	Some PDA features	Marked PDA features	
PART 3 - SECTION XV – IMAGINATION					
Role play – lacks ability to pretend with awareness of dramatic role.	N=150	83	81	82	p > .1
No pretend or role play of any sort	N=151	33	44	27	p > .1
Role play lacks spontaneous quality – is copied	N=100	56	38	64	p > .1
Absence of shared imaginative activities with age peers.	N=99	57	44	64	p > .1
Lack of curiosity about self or the world	N=153	74	88	82	p > .1
Lack of spontaneous pretend play	N=149	47	44	36	p > .1
Repetitive pretend play – not varied	N=81	44	56	45	p > .1
Repetitive acting out roles	N=72	51	86	83	p = .093
PART 4 - SECTION V - ROUTINES & RESISTANCE TO CHANGE					
Repetitive questionning	N=147	56	88	55	p > .1
Repetitive themes	N=141	72	81	73	p > .1
Obsession with a person	N=152	32	25	36	p > .1
PART 4 - SECTION VI - OVERALL PATTERN OF ACTIVITIES					
**Inability to remain sitting**	**N=147**	**32**	**63**	**55**	**p = .008**
Continual motor restlessness	N=150	41	63	45	p > .1
Hyperactivity	N=151	37	44	55	p > .1
**Fixed, repeated motor stereotypies**	**N=153**	**29**	**75**	**27**	**p = .012**
Excessive repetition of activities	N=153	27	38	18	p > .1
PART 5 - SECTION I – EMOTIONS					
Lack of emotional expression	N=151	55	56	64	p > .1
Unhappiness, misery	N=153	52	56	82	p > .1
**Changeable mood**	N=153	58	81	73	p = .080
**Crying and moaning**	**N=152**	**23**	**44**	**45**	**p = .031**
**Laughing for no reason**	**N=152**	**42**	**56**	**91**	**p = .010**
Puzzlement	N=151	61	63	45	p >.1
Anxiety	N=153	75	94	91	p = .069
Special Fears	N=151	52	38	55	p > .1

Items marked in bold reached the nominal significance threshold for increased endorsement in the PDA groups vs. the rest of the sample (Fisher's exact test, two sided).

		% MINOR OR MARKED			PDA vs. Rest of the sample (Fisher's exact test, 2 sided)
		Rest of sample	Some PDA features	Marked PDA features	
PART 6 - SECTION I - BEHAVIOUR AFFECTING OTHERS					
Wandering	N=150	33	50	55	p > .1
**Destructiveness**	**N=152**	**30**	**63**	**73**	**p < .001**
**Noisiness**	**N=151**	**27**	**69**	**55**	**p < .001**
**Temper tantrums**	**N=152**	**62**	**88**	**91**	**p = .006**
**Physical aggression**	**N=151**	**53**	**88**	**100**	**p < .001**
**Anger towards parents**	**N=149**	**48**	**88**	**82**	**p < .001**
**Blaming other people**	**N=149**	**43**	**94**	**100**	**p < .001**
**Harassment of others**	**N=150**	**11**	**38**	**70**	**p < .001**
**Behaviour in public places**	**N=153**	**46**	**69**	**82**	**p = .010**
**Personal modesty**	**N=153**	**26**	**63**	**36**	**p = .012**
**Psychological barriers**	**N=153**	**41**	**75**	**91**	**p < .001**
**Approaching strangers**	**N=153**	**24**	**56**	**64**	**p < .001**
**Embarrassing remarks in public**	**N=141**	**59**	**81**	**90**	**p = .009**
**Interrupting conversations**	**N=138**	**75**	**93**	**100**	**p = .029**
**Inappropriate response to others' emotions**	**N=153**	**48**	**88**	**91**	**p < .001**
**Difficult or objectionable personal habits**	**N=153**	**29**	**56**	**73**	**p = .001**
**Scatters or throws objects around**	**N=152**	**20**	**50**	**64**	**p < .001**
**Lack of co-operation**	**N=152**	**62**	**94**	**100**	**p < .001**
**Needs constant supervision**	**N=152**	**44**	**81**	**100**	**p < .001**
**Apparently manipulative behaviour**	**N=152**	**42**	**94**	**100**	**p < .001**
**Demands carer's attention**	**N=151**	**65**	**94**	**100**	**p < .001**
**Difficulties with other people**	**N=150**	**21**	**69**	**100**	**p < .001**
**Socially shocking behaviour**	**N=152**	**7**	**38**	**73**	**p < .001**
**Fantasising, lying, cheating, stealing**	**N=150**	**25**	**50**	**80**	**p < .001**

Items marked in bold reached the nominal significance threshold for increased endorsement in the PDA groups vs. the rest of the sample (Fisher's exact test, two sided).
